# Disparities in Access to Mental Health Services Among Children Diagnosed with Anxiety and Depression in the United States

**DOI:** 10.1007/s10597-024-01305-3

**Published:** 2024-06-22

**Authors:** Asos Mahmood, Satish Kedia, Hassan Arshad, Xichen Mou, Patrick J. Dillon

**Affiliations:** 1 Center for Health System Improvement, College of Medicine, University of Tennessee Health Science Center, Memphis, TN USA; 2 Department of Medicine-General Internal Medicine, College of Medicine, University of Tennessee Health Science Center, 956 Court Ave Avenue, Ste D222A, Memphis, TN 38103 USA; 3 Division of Social and Behavioral Sciences, School of Public Health, The University of Memphis, Memphis, TN USA; 4 Division of Epidemiology, Biostatistics, Environmental Health, School of Public Health, The University of Memphis, Memphis, TN USA; 5 School of Communication Studies, Kent State University at Stark, North Canton, OH USA

**Keywords:** Children and adolescents, Mental health disorder, Depression, Anxiety, Disparities, Mental health services, Health care access

## Abstract

Child and adolescent mental health are major public health concerns in the US. Overall, 20% of US children have a reported mental health condition, while an estimated 40% will be diagnosed with one by age 18. Despite these concerns, little is known about factors associated with access to mental health services among children and adolescents. We analyzed data from a sample of 6655 children (aged 6 to 17 years) with either anxiety and/or depression drawn from the 2020–2021 National Survey of Children’s Health (NSCH). A multivariable logistic regression model was fit to investigate predisposing, enabling, and need factors associated with caregiver’s (i.e., parent or other guardian) perceived access to mental health services for their children. Approximately 50.8% of caregivers perceived obtaining mental health services for their children to be somewhat difficult, very difficult, or impossible. Children meeting criteria for having a medical home had lower odds of experiencing such difficulties (adjusted [a]OR = 0.38; 95% CI: 0.30–0.49). Further, compared to children who sometimes or never had health insurance coverage for mental or behavioral health needs, children who were always insured (aOR: 0.19; 95% CI 0.14, 0.25) and those who usually had coverage (aOR: 0.38; 95% CI 0.28, 0.51) had lower odds of experiencing perceived difficulties in obtaining care. The results indicate several enabling and need predictors of perceived access to mental health services––highlighting potential structural barriers to care access. Efforts to address access challenges should adopt a multifaceted approach and be tailored to families living in poverty, those with limited health coverage, and minoritized children with less than optimal general health.

**Supplementary Information:**

The online version contains supplementary material available at 10.1007/s10597-024-01305-3.

## Introduction

Mental health status of children and adolescents is a public health concern and an essential indicator of populations’ health and wellbeing (U.S. Department of Health and Human Services, n.d.). Mental health conditions such as anxiety and depression are among the most common psychiatric disorders among all pediatric age groups (Walter et al., 2020, 2023). Child and adolescent mental health, and the higher prevalence of anxiety and depression, have garnered increasing attention across the United States (US) in recent years (Bitsko et al., 2022; Lebrun-Harris et al., 2022; Shim et al., 2022). An analysis of US surveillance data collected between 2013 and 2019 suggests that approximately one in 11 children (9.4%–9.8%) between ages three and 17 experienced anxiety and attention-deficit/hyperactivity disorder; the same analysis indicates that around one-fifth (20.9%) of children aged 12–17 years reportedly experienced at least one major depressive episode during the study period (Bitsko et al., 2022). Similar national statistics indicate that rates of anxiety and depression among children aged three to 17 years increased by 29% and 27% respectively between 2016 and 2020 (Lebrun-Harris et al., 2022). Overall, 20% of US children have an identified mental health condition, while an estimated 40% will meet the criteria for one by the time they turn 18 (Bitsko et al., 2022; Shim et al., 2022).

To ensure children’s health and wellbeing and improve quality of life in this critical developmental stage, proper access to essential healthcare services, including mental health services, is paramount (Office of the Surgeon General, 2021). Proactively addressing potential barriers to mental health care access is especially important for children and adolescents with anxiety and depression. The association between poverty, mental health incidence, and service access/outcomes, both in children and adults, has been previously explored in the literature. Food insecurity, housing instability, residing in unsafe neighborhoods, and insufficient or inconsistent income are all stressors that adversely contribute to an increased risk of mental health disorders (Myers, 2020; Padgett, 2020; Sareen et al., 2011). There is a proposed bidirectional causal relationship between poverty and mental health conditions (Anakwenze & Zuberi, 2013; Ten Have et al., 2021). Loss of income or substandard housing, for example, can contribute to depression by perpetuating anxiety and uncertainties about finances or expose residents to environmental stressors such as violence, pollution, and a lack of walkable neighborhoods or areas for recreation, all of which can negatively impact mental wellbeing (Berke et al., 2007; Borg et al., 2021; McDonald & Richmond, 2008; Newbury et al., 2021).

Despite the high rates of anxiety and depression, relatively small numbers of US children and adolescents receive treatment for these conditions. For example, only 10.1% (9.8%–10.5%) of children and adolescents aged three to 17 years, regardless of diagnoses of the mental condition, received any treatment or counseling from a mental health professional between 2016 and 2019 (Bitsko et al., 2022). Furthermore, during the same period, only 7.8% of those in this age group were prescribed medication for difficulties with managing emotions, concentrating, or regulating behavior (Bitsko et al., 2022). The data further demonstrated that children in the US from families of lower socioeconomic status as well as those from minority populations were more prone to mental health conditions and had less access to mental health care than their peers (Bitsko et al., 2022; Shim et al., 2022). Though there are no consistent reported patterns in access to mental health services across family poverty levels, the available figures largely support previous studies indicating people from households with lower socioeconomic status are less likely to visit healthcare providers (Cree et al., 2018; Hodgkinson et al., 2017; Mojtabai, 2021) and more likely to have unmet mental health needs (Kataoka et al., 2002; Nagata et al., 2022; Santiago et al., 2013). In general, people from low-income backgrounds are at a greater risk of having unmet mental health needs (Kataoka et al., 2002; Nagata et al., 2022) and face logistical or systemic barriers to obtaining mental health services (Santiago et al., 2013). Similarly, studies have long suggested that people from minoritized groups are less likely to access mental health services, even after controlling for factors such as socioeconomic and health status (Alegria et al., 2010; Dobalian & Rivers, 2008; Zahner & Daskalakis, 1997). For example, while approximately 12% of White children accessed mental health treatment between 2013 and 2019, treatment rates were less than 10% for Black (9.8%) and Latinx (8.7%) children (Bitsko et al., 2022).

High rates of mental health conditions (predominantly anxiety and depression), coupled with concerns regarding access to appropriate treatment, have led scholars and national healthcare organizations to declare a “state of emergency” pertaining to child and adolescent mental health (e.g., American Academy of Pediatrics, 2021; Shim et al., 2022). In 2021, the US Surgeon General issued a public health advisory and call to action emphasizing the importance of addressing the urgent public health crisis of mental health among children and adolescents, especially in the wake of COVID-19 (Office of the Surgeon General, 2021). The advisory included practical points of action for all stakeholders, including an outline of subjects for continuing research, including a priority on data pertaining to at-risk youth populations (Office of the Surgeon General, 2021). There is a need to better understand potential barriers and facilitators to mental health care access among US children and adolescents, especially those from low-income households and minoritized communities (Office of the Surgeon General, 2021). Although several barriers to mental health treatment (e.g., financial hardship, health insurance status, and stigma pertaining to mental illness and help-seeking) are well documented among adult populations (see e.g., Coombs et al., 2021; Leong & Kalibatseva, 2011), there are, to our knowledge, limited studies examining factors that impact access to mental health care among children and adolescents.

In the present study, we employ Andersen’s Behavioral Model of Health Services Use (BMHSU) to identify barriers and facilitators that influence perceived access to mental health services among children and adolescents with anxiety and/or depression (R. Andersen, 1968; R. M. Andersen, 1995; Phillips et al., 1998). The BMHSU model focuses on factors that impact health service access and utilization; these factors are organized into three primary categories: predisposing factors, enabling factors, and need factors. Predisposing factors include demographic variables such as age, sex, and race/ethnicity. Enabling factors include logistical aspects that impact people or patients’ access to care such as residential location, people in their household, income, and insurance coverage. The final category, need factors, encompasses a person’s current health status, as captured by self-assessments and professional assessments; these assessments are deemed equally valuable in providing insights into reasons for seeking care and the type of care that is needed, respectively (Andersen, 1995).

The BMHSU has been utilized in numerous studies regarding health services use since its conception; its applications include studies in the utilization of psychiatric (Dhingra et al., 2010; Fortin et al., 2018), primary care (Alhalaseh et al., 2022), and dental (Song et al., 2021) services, among others. In this study, we draw upon the BMHSU model to identify predisposing, enabling, and need-for-care factors pertaining to perceived access to mental health services among US children and adolescents with anxiety and/or depression. We do so by analyzing data from the 2020 and 2021 waves of the National Survey of Children’s Health (NSCH).

## Methods

### Data Source and Study Sample

This study utilized cross-sectional data obtained from the 2020 and 2021 cycles of the NSCH. This national survey is funded and directed by the Health Resources and Services Administration’s Maternal and Child Health Bureau and fielded by the US Census Bureau (Child & Adolescent Health Measurement Initiative, 2021b). It is administered both online and by mail. Potential survey respondents are selected through a random selection of addresses across the US; some are mailed instructions to access the online version of the survey while others are also given a paper copy to complete either upon initial notification or after unsuccessful attempts to complete the online questionnaire (Child & Adolescent Health Measurement Initiative, 2021b). Initial screening involves asking parents or guardians to provide age and sex of all children in the household to stratify for at least one child between the ages of zero to 17 residing in the household. One child from each household is then randomly selected to be the subject of the topical questionnaire. Parents or guardians are asked to complete one of three versions of the survey, which are divided into separate age ranges (i.e., 0–5 years, 6–11 years, and 12–17 years).

The NSCH contains a variety of questions addressing children’s physical and mental health as well as their access to health services, experiences with health care providers, health insurance, schooling, and extracurricular activities, in addition to family relationships, finances, and dynamics. Data are usually collected from an adult member within the household who has knowledge about the selected child’s health. A combined total of 93,669 respondents completed NSCH surveys during 2020 and 2021 (42,777 in 2020 and 50,892 in 2021). The weighted overall response rate was 42.4% for 2020 and 40.3% for 2021 (Child and Adolescent Health Measurement Initiative, 2021c). The final sample for this study included 6655 children aged six to 17 years with an affirmative caregiver-reported diagnosis of either anxiety and/or depression and no missing items for the dependent variable. The study’s emphasis and its analytical sample focused on those with anxiety and depression because they are the most prevalent mental health disorders among this age group (Lebrun-Harris et al., 2022).

For this study, the final sample was identified by applying four filters to the combined 2020–2021 NSCH dataset. First, children who are currently diagnosed with anxiety and/or depression, as reported by a parent or guardian were included. Second, due to scarcity of reported mental health diagnoses in this age range, children under age six were excluded (Child & Adolescent Health Measurement Initiative, 2021a; United States Census Bureau, 2021). Third, children who did not need to see a mental health professional or receive mental health care treatment or counseling during the past 12 months, based on parents’/guardians’ negative response to the survey question, “*DURING THE PAST 12 MONTHS, has this child received any treatment or counseling from a mental health professional?*” were excluded. The rationale behind this exclusion was that this group of children’s parents/guardians were not asked about accessing mental health services and hence did not share their opinions on this issue (see the dependent variable). Lastly, children with missing data on the dependent variable of mental health treatment access (n = 71) were excluded.

### Dependent Variable

The study’s dependent variable assessed whether a child experienced difficulty in obtaining mental health services (henceforth “difficulty in access”). It was analyzed based on a survey participant’s (i.e., a parent or guardian’s) response to the following question: “*How difficult was it to get the mental health treatment or counseling that this child needed?*” The response options included (1) “not difficult”, (2) “somewhat difficult”, (3) “very difficult”, and (4) “not possible to obtain care.” We re-coded responses into a binary outcome regarding difficulties accessing mental health services as being either “not difficult” to obtain or “somewhat/very difficult or not possible” to obtain.

### Explanatory Variables

According to the BMHSU framework, an individual’s propensity to utilize healthcare services is influenced by aspects within three dynamics. In the category of predisposing factors are innate characteristics such as one’s age, sex, and race or ethnicity. Based on Andersen’s Model, four variables in the present study were identified as predisposing factors: the child’s age (6–11 years and 12–17 years), biological sex (male vs. female), race and ethnicity (non-Hispanic White, non-Hispanic Black, Hispanic, non-Hispanic Asian and others), and family structure (two parents [Currently married or unmarried], single parent [mother or father], grandparent household or other). Seven characteristics were identified as enabling factors including the highest level of education obtained by someone residing in the household (less than high school, high school graduate, some college, and college graduate or more), household poverty status (< 100%, 100–199%, 200–299%, 300–399%, ≥ 400% of the federal poverty level [FPL]), having a child who meets criteria for having a medical home (yes vs. no) which is defined as a high-quality primary care model that is accessible, family-centered, continuous, comprehensive, coordinated, compassionate, and culturally effective (American Academy of Pediatrics, n.d.-a), consistency of insurance coverage (continuously covered by any insurance over the last 12 months or having gaps in coverage/not insured in the past 12 months), frequency that health insurance covers services for child’s mental or behavioral health needs (always, usually, sometimes, or never), whether the child had a usual place for health care/medical advice (yes vs. no), and primary household language (English vs. other than English).

Finally, need factors concern the individual’s perceptions of their abilities and overall health status. Need factors in this study were divided into those pertaining to parents/caregivers and the child. Caregivers were asked to rate the state of their physical and mental or behavioral health (good vs. not good). Need factors for children included asking about the child’s state of overall health (excellent or very good, good, fair, or poor)*.* Further, despite having children in the analytical sample who were diagnosed with both anxiety and depression (i.e., comorbid anxiety and depression), some children had one condition but not the other. Therefore, we incorporated two condition-specific factors, as child need factors, indicating whether a doctor had told the caregiver their child had anxiety (yes vs. no) or depression (yes vs. no).

### Statistical Analyses

Raw frequencies and weighted proportions of explanatory and dependent variables were first computed (see Table 1) and two-way percentages were analyzed (see Table 2). All weighted percentages were calculated based on the sampling weights that are available with the dataset to generate nationally representative, population-level proportions in the US. Further, all values computed in this study are nationally representative of children diagnosed with anxiety and/or depression, as reported by their parent or guardian. In the NSCH dataset, the sampling weight approximately equals the inverse of the probability of selection and is further adjusted by multiple factors including nonresponse, number of children in households, stratification, etc. Since the analyses were performed on combined 2020 and 2021 NSCH data, we used the adjusted final weights, available with the dataset, that accounts for combining two years of data (US Census Bureau, 2023). Logistic regression models were fit to examine the association between three groups of predictive factors (i.e., predisposing, enabling, and need), guided by Andersen’s behavioral model, and perceived difficulty in access. These results are summarized in Table 3. Model 1 regressed the dependent variable solely on each independent factor, which yields crude odds ratios (ORs) and 95% confidence intervals (CIs); Model 2 presented adjusted (a) ORs by fitting a multivariable logistic regression adjusted for all three groups of predictive factors. We similarly accounted for the complex design features of the NSCH in the regression models to generate population-representative estimates. Additional subgroup analyses were performed by assessing associations among the subsample of children who were diagnosed with anxiety only, given that it is the most common mental health condition among children, and an additional analysis for the subsample of children who were diagnosed with both anxiety and depression (i.e., comorbid anxiety and depression). Data analysis was conducted employing R software (version 4.2.1). Specifically, the R “survey” package was used to account for the complex survey features of the NSCH. The statistical significance threshold was set at *P*-value < 0.05.

**Table 1:** Sociodemographic and other characteristics of children with anxiety and/or depression (NSCH, n = 6,655; 2020–2021, the United States)

	Children with Anxiety and/or Depression	
Characteristics	Frequency	weighted % (95% CI)^a^
Predisposing factors		
Age groups (years)		
6 to 11	1848	29.5 (27.0, 32.0)
12 to 17	4807	70.5 (68.0, 73.0)
Sex		
Male	2969	43.9 (41.2, 46.6)
Female	3686	56.1 (53.4, 58.8)
Race and ethnicity		
Hispanic	755	22.7 (19.6, 25.8)
White, non-Hispanic	4892	58.3 (55.3, 61.3)
Black, non-Hispanic	314	10.7 (8.6, 12.7)
Other/Multi-racial, non-Hispanic	694	8.3 (6.9, 9.7)
Family structure		
Two parents (Currently married or unmarried)	4238	63.3 (60.5, 66.0)
Single parent (mother or father)	1823	29.6 (26.9, 32.3)
Grandparent household or other	449	7.2 (6.0, 8.3)
Enabling Factors		
Highest household education level		
Less than high school	108	6.9 (4.3, 9.4)
High school graduate^b^	816	16.9 (14.8, 19.0)
Some college	1616	22.9 (20.7, 25.1)
College graduate or more	4115	53.3 (50.5, 56.1)
Primary household language		
English	6453	92.4 (89.9, 95.0)
Other than English	172	7.6 (5.0, 10.1)
Household poverty status		
0–99% FPL	845	15.8 (13.5, 18.0)
100–199% FPL	1153	21.6 (19.2, 24.0)
200–399% FPL	2014	30.6 (27.9, 33.2)
≥ 400% FPL	2643	32.1 (29.7, 34.4)
Medical home^c^		
Yes	2666	37.0 (34.4, 39.6)
No	3989	63.0 (60.4, 65.6)
Consistency of insurance coverage		
Insured all past 12 months	6314	93.8 (92.0, 95.6)
Currently uninsured or had gaps in coverage in the past 12 months	311	6.2 (4.4, 8.0)
Insurance coverage for mental or behavioral health needs		
Always	2870	43.5 (40.8, 46.3)
Usually	1858	26.9 (24.5, 29.2)
Sometimes or never	1556	22.0 (20.0, 24.0)
Uninsured	325	7.6 (5.6, 9.7)
Usual place for health care/advice		
Yes	5897	85.0 (82.6, 87.3)
No	746	15.0 (12.7, 17.4)
Caregiver need factors		
Caregiver’s self-rated physical health status		
Good	5683	83.8 (81.4, 86.2)
Not Good	805	16.2 (13.8, 18.6)
Caregiver’s self-rated mental or emotional health		
Good	5468	82.2 (79.8, 84.6)
Not Good	1018	17.8 (15.4, 20.2)
Child Need factors		
Child has anxiety		
No	494	9.8 (7.7, 11.9)
Yes	6113	90.2 (88.1, 92.3)
Child has depression		
No	3250	48.8 (46.0, 51.5)
Yes	3390	51.2 (48.5, 54.0)
Child has behavioral or conduct problems		
No	4320	61.5 (58.7, 64.2)
Yes	2296	38.5 (35.8, 41.3)
Child has ADHD		
No	3884	58.3 (55.6, 61.0)
Yes	2730	41.7 (39.0, 44.4)
General health status of child		
Excellent or very good	4878	69.6 (66.8, 72.4)
Good	1407	22.1 (19.7, 24.5)
Fair or poor	363	8.3 (6.1, 10.4)
Study outcome		
Difficulties obtaining mental health care		
Not difficult	3221	49.2 (46.5, 52.0)
Somewhat difficult/very difficult or not possible to obtain care	3434	50.8 (48.0, 53.5)

*FPL* federal poverty level; *ADHD* attention-deficit/hyperactivity disorder

^a^Frequencies represent unweighted sample frequencies while percentages are national-level estimates of values

^b^Including vocational, trade, or business schools

^c^Whether the child meets the criteria for having a medical home

**Table 2:** Sociodemographic and other characteristics of children with anxiety and/or depression by levels of perceived difficulty accessing mental health care services (NSCH, n = 6,655; 2020–2021, the United States)

	Difficulties obtaining mental health care				
	Not Difficult (n = 3,221)		Somewhat difficult/very difficult or not possible (n = 3,434)		
Characteristics	Frequency	weighted % (95% CI)^a^	Frequency	weighted % (95% CI)^a^	*P*-value
Predisposing factors					
Age groups (years)					
6 to 11	837	27.6 (23.9, 31.2)	1011	31.4 (27.9, 34.9)	0.002
12 to 17	2384	72.4 (68.8, 76.1)	2423	68.6 (65.1, 72.1)	
Sex					
Male	1399	42.8 (38.9, 46.6)	1570	45.1 (41.2, 48.9)	0.06
Female	1822	57.2 (53.4, 61.1)	1864	54.9 (51.1, 58.8)	
Race and ethnicity					
Hispanic	347	24.2 (19.4, 28.9)	408	21.3 (17.3, 25.3)	0.14
White, non-Hispanic	2382	57.8 (53.5, 62.0)	2510	58.8 (54.6, 62.9)	
Black, non-Hispanic	167	10.0 (7.8, 12.2)	147	11.3 (7.9, 14.7)	
Other/Multi-racial, non-Hispanic	325	8.0 (6.1, 9.9)	369	8.6 (6.6, 10.6)	
Family structure					
Two parents (currently married or unmarried)	2043	63.0 (59.2, 66.9)	2195	63.4 (59.5, 67.4)	0.003
Single parent (mother or father)	843	28.6 (24.8, 32.3)	980	30.5 (26.6, 34.4)	
Grandparent household or other	248	8.4 (6.6, 10.1)	201	6.1 (4.6, 7.5)	
Enabling Factors					
Highest household education level					
Less than high school	65	8.2 (4.4, 12.1)	43	5.6 (2.1, 9.0)	< 0.001
High school graduate^b^	443	18.1 (15.4, 20.9)	373	15.8 (12.5, 19.0)	
Some college	807	22.9 (19.8, 26.1)	809	22.9 (19.8, 26.0)	
College graduate or more	1906	50.7 (46.7, 54.7)	2209	55.8 (51.8, 59.8)	
Primary household language					
English	3116	91.8 (87.9, 95.6)	3337	93.0 (89.6, 96.4)	0.51
Other than English	88	8.2 (4.4, 12.1)	84	7.0 (3.6, 10.4)	
Household poverty status					
0–99% FPL	438	15.3 (12.8, 17.9)	407	16.2 (12.5, 19.9)	0.19
100–199% FPL	557	21.9 (18.4, 25.5)	596	21.3 (18.1, 24.4)	
200–399% FPL	957	32.2 (28.2, 36.2)	1057	29.0 (25.6, 32.4)	
≥ 400% FPL	1269	30.5 (27.4, 33.7)	1374	33.5 (30.2, 36.8)	
Medical home^c^					
Yes	1692	48.5 (44.5, 52.4)	974	25.9 (22.5, 29.3)	< 0.001
No	1529	51.5 (47.6, 55.5)	2460	74.1 (70.7, 77.5)	
Consistency of insurance coverage					
Insured all past 12 months	3083	96.0 (94.7, 97.3)	3231	91.6 (88.3, 95.0)	0.002
Currently uninsured or had gaps in coverage in the past 12 months	123	4.0 (2.7, 5.3)	188	8.4 (5.0, 11.7)	
Insurance coverage for mental or behavioral health needs					
Always	1881	58.5 (54.6, 62.4)	989	29.1 (25.4, 32.8)	< 0.001
Usually	781	25.5 (21.8, 29.2)	1077	28.1 (25.1, 31.2)	
Sometimes or never	405	10.8 (8.8, 12.8)	1151	32.8 (29.5, 36.2)	
Uninsured	133	5.2 (3.6, 6.8)	192	10.0 (6.3, 13.6)	
Usual place for health care/advice					
Yes	2829	85.4 (82.7, 88.0)	3068	84.6 (80.8, 88.4)	0.08
No	384	14.6 (12.0, 17.3)	362	15.4 (11.6, 19.2)	
Caregiver Need Factors					
Caregiver’s self-rated physical health status					
Good	2768	84.0 (80.5, 87.6)	2915	83.6 (80.3, 86.9)	0.02
Not Good	355	16.0 (12.4, 19.5)	450	16.4 (13.1, 19.7)	
Caregiver’s self-rated mental or emotional health					
Good	2732	85.5 (82.7, 88.3)	2736	79.0 (75.2, 82.7)	< 0.001
Not Good	391	14.5 (11.7, 17.3)	627	21.0 (17.3, 24.8)	
Child Need factors					
Child has anxiety					
No	259	11.4 (7.9, 15.0)	235	8.1 (5.8, 10.5)	0.07
Yes	2938	88.6 (85.0, 92.1)	3175	91.9 (89.5, 94.2)	
Child has depression					
No	1700	52.8 (48.9, 56.7)	1550	44.9 (41.1, 48.7)	< 0.001
Yes	1514	47.2 (43.3, 51.1)	1876	55.1 (51.3, 58.9)	
Child has behavioral or conduct problems					
No	2247	66.7 (63.0, 70.3)	2073	56.4 (52.5, 60.3)	< 0.001
Yes	961	33.3 (29.7, 37.0)	1335	43.6 (39.7, 47.5)	
Child has ADHD					
No	1927	60.0 (56.2, 63.8)	1957	56.7 (52.8, 60.5)	0.012
Yes	1268	40.0 (36.2, 43.8)	1462	43.3 (39.5, 47.2)	
General health status of child					
Excellent or very good	2486	73.3 (69.4, 77.2)	2392	66.1 (62.1, 70.1)	< 0.001
Good	589	18.8 (15.6, 21.9)	818	25.3 (21.8, 28.8)	
Fair or poor	142	7.9 (4.8, 11.0)	221	8.6 (5.6, 11.6)	

*FPL* federal poverty level; *ADHD* attention-deficit/hyperactivity disorder

^a^Frequencies represent unweighted sample frequencies while percentages are national-level estimates of values

^b^Including vocational, trade, or business schools

^c^Whether the child meets the criteria for having a medical home

**Table 3:** Logistic regressions for children diagnosed with anxiety and/or depression, modeling the associations between predisposing, enabling, and need factors of perceived access to child’s mental health services according to caregivers (n = 6655, NSCH, 2019–2020, the United States)

	Model 1	Model 2
Characteristics	Crude OR (95% CI)	Adjusted OR (95% CI)
Predisposing Factors		
Age groups (ref: 6–11 years)		
12–17 years	0.83 (0.65, 1.06)	0.77 (0.58, 1.00)
Sex (ref: female)		
Male	1.10 (0.88, 1.37)	1.11 (0.88, 1.41)
Race and ethnicity (ref: non-Hispanic White)		
Hispanic	0.87 (0.61, 1.23)	0.94 (0.65, 1.38)
Non-Hispanic Black	1.11 (0.73, 1.68)	1.31 (0.85, 2.00)
Non-Hispanic Asian and others	1.05 (0.74, 1.50)	0.94 (0.64, 1.40)
Family structure (ref: single parent)		
Two parents (currently married or unmarried)	0.94 (0.72, 1.23)	1.00 (0.76, 1.32)
Grandparent household or other	0.68 (0.46, 1.00)	0.77 (0.51, 1.16)
Enabling Factors		
Highest household education level (ref: college graduate or more)		
Less than high school	0.61 (0.27, 1.41)	0.40 (0.17, 0.96)*
High school^a^	0.79 (0.58, 1.07)	0.74 (0.49, 1.10)
Some college	0.91 (0.71, 1.17)	0.85 (0.63, 1.15)
Primary household language (ref: English)		
Other than English	0.84 (0.40, 1.74)	1.35 (0.54, 3.34)
Household poverty status (ref: < 99% FPL)		
100–199% FPL	0.92 (0.61, 1.37)	0.94 (0.63, 1.39)
200–399% FPL	0.85 (0.59, 1.24)	0.85 (0.56, 1.32)
≥ 400% FPL	1.04 (0.73, 1.47)	1.02 (0.66, 1.56)
Medical home (ref: no)^b^	0.37 (0.29, 0.47)‡	0.38 (0.30, 0.49)‡
Consistency of insurance coverage (ref: currently uninsured or had gaps in coverage in the past 12 months)		
Insured all past 12 months	0.45 (0.26, 0.79)†	0.95 (0.50, 1.80)
Insurance coverage for mental or behavioral health needs (ref: sometimes or never)		
Always	0.16 (0.12, 0.22)‡	0.19 (0.14, 0.25)‡
Usually	0.36 (0.27, 0.49)‡	0.38 (0.28, 0.51)‡
Uninsured	0.63 (0.36, 1.09)	0.67 (0.34, 1.30)
Usual place for health care/advice (ref: no)		
Yes	0.94 (0.66, 1.36)	1.28 (0.85, 1.92)
Caregiver Need Factors		
Caregiver’s self-rated physical health status (ref: not good)		
Good	0.97 (0.68, 1.39)	1.06 (0.74, 1.50)
Caregiver’s self-rated mental or emotional health (ref: not good)		
Good	0.64 (0.46, 0.88)†	0.77 (0.55, 1.08)
Child Need Factors		
General health status of child (ref: fair or poor)		
Excellent or very good	0.83 (0.47, 1.47)	0.88 (0.54, 1.44)
Good	1.24 (0.67, 2.29)	1.30 (0.75, 2.23)
Child has anxiety (ref: no)	1.46 (0.92, 2.33)	1.84 (1.14, 2.96)*
Child has depression (ref: no)	1.37 (1.10, 1.71)†	1.87 (1.45, 2.42)‡

*OR* odds ratio; *CI* confidence interval; *ref* reference; *FPL* federal poverty level

^*^p-value < 0.05; †p-value < 0.01; ‡p-value < 0.001

Model 1: crude odds ratio

Model 2: adjusted for predisposing, enabling, and need factors

^a^Including vocational, trade, or business schools

^b^Whether the child meets the criteria for having a medical home

## Results

### General Characteristics of Children Diagnosed with Anxiety and/or Depression

Our analytical sample included 6,655 US children and adolescents (aged 6 to 17 years) diagnosed with anxiety and/or depression, as reported by their parent/guardian. The sample represents 41.1 million children across two survey years in the country. Those aged six to 11 years comprised a third of the sample (29.5%) while those aged 12 to 17 made up the rest of the children (70.5%). Males and females each made up about 43.9% and 56.1% of the children, respectively. The majority of children with anxiety and/or depression were non-Hispanic Whites (58.3%). Hispanic and non-Hispanic Black children accounted for approximately 22.7% and 10.7% of these diagnoses, respectively. For almost two-thirds (63.3%) of children the family structure was composed of two parents (married or unmarried). More than half the sample came from households where at least one caregiver had a college degree or higher (53.3%), and the vast majority identified English as the primary language in their homes (92.4%). Nearly one-third of households had an income at or above 400% of the FPL (32.1%), and those standing at 0–99% FPL and 100–199% FPL made about 15.8% and 21.6%, respectively. Further, about two-thirds of the children met criteria for having a medical home in the past year (63.0%). More than 90% of children (93.8%) had consistent health coverage during the past year. Over two-thirds (70.4%) of respondents reported that their child’s health insurance always or usually offered benefits or covered services for mental or behavioral health needs. Further, about 85.0% of children had a usual place for health care/ medical advice (see Table 1).

The majority of children’s caregivers self-reported being in “good” health both physically (83.8%) and mentally (82.2%). More than 90% of children had been told by a doctor that they had anxiety (90.2%) while more than half were told they had depression (51.2%). In addition, more than one-third had been told they had behavioral or conduct problems (38.5%) while over 41.7% had received an ADHD diagnosis. Finally, regarding general health, 69.6% of children were reported as being in excellent or very good health (See Table 1).

### Cross-Tabulated Associations of Children Characteristics with Parents’ Perceived Access to Mental Health Services

Caregiver perceptions about the difficulty in access found that 50.8% reported somewhat or very difficult to obtain mental health care, or that it had been impossible to receive care altogether (see Table 1). Among this subgroup, 68.6% were children aged 12 to 17 years, 54.9% females, 58.8% non-Hispanic Whites, and 63.4% had a family structure of two parents (see Table 2). Moreover, among the subgroup of perceived difficulty in access, 55.8% were children from households where at least one caregiver had a college degree or higher and 33.5% of households had an income at or above 400% of the FPL. Full results of two-way analyses are provided in Table 2.

### Factors Associated with Perceived Access to Mental Health Services

Our multivariable logistic regression analysis results are provided in Table 3. Several enabling factors were significantly associated with difficulties accessing mental health services among children with anxiety and/or depression. Children from households where the highest education level was less than a high school diploma had less perceived difficulty obtaining access when compared to households with college graduate or more in education (aOR: 0.40; 95% CI: 0.17, 0.96). Additionally, children who met criteria for having a medical home in the past year had about 62% lower odds of experiencing such difficulties (aOR: 0.38; 95% CI: 0.30, 0.49) compared to children who did not. Furthermore, our analyses revealed that, compared to children who sometimes or never had health insurance coverage for mental or behavioral health needs, children who were always insured for such needs had 81% lower odds of experiencing difficulties obtaining care (aOR: 0.19; 95% CI 0.14, 0.25) and those who usually had coverage had 62% lower odds of perceived difficulty in access (aOR: 0.38; 95% CI 0.28, 0.51) (see Table 3)*.* Finally, children with reported anxiety (aOR: 1.84; 95% CI 1.14, 2.96) and those with depression (aOR: 1.87; 95% CI 1.45, 2.42) as standalone conditions had 84% and 87% higher odds of perceived difficulty in access, respectively, compared to corresponding children without the condition (see Table 3).

Results from the subgroup analyses of children with anxiety only, and children with comorbid anxiety and depression show almost identical patterns of associations with children’s predisposing, enabling, and need factors in terms of perceived access to mental health care services, per parent/guardian report (see Supplementary Tables 1 and 2). Of note, among the subgroup of children with comorbid anxiety and depression, non-Hispanic Blacks (aOR: 4.18; 95% CI 2.13, 8.23) and non-Hispanic Asians and others (aOR: 1.60; 95% CI 1.02, 2.54) had significantly higher odds of perceived difficulty in gaining access to needed services when compared to non-Hispanic White children (see Supplementary Table 2).

## Discussion

The results obtained in the current study explore a unique aspect of access to mental health care among children with anxiety and/or depression and will add to the burgeoning scientific evidence in the field. Our findings demonstrate several notable trends indicating factors that were significantly associated with perceived barriers and facilitators to mental health service access among children with anxiety and/or depression, as reported by their parents/guardians. Among potential enabling factors of perceived access, household educational level, the child meeting criteria for having a medical home, and insurance coverage for mental or behavioral health needs were significant predictors of perceived access to mental health care services. Among the subgroup of children with comorbid anxiety and depression, the results showed child’s race and ethnicity are a predisposing factor for perceived difficulty accessing mental health care. Being a member of non-Hispanic Black or non-Hispanic Asian and other minoritized populations was significantly associated with higher perceived difficulty accessing mental health care services. These findings are collectively in line with several other studies in this domain which report that parental education, health insurance coverage, race and ethnicity, and various socioeconomic factors determine the need for, and actual access to, mental health treatment among children and adolescents with mental health care needs (Cummings & Druss, 2011; Flores & Research, 2010; Rodgers et al., 2022; Triplett et al., 2022).

In general, evidence indicates that the medical home model of care leads to improved quality of life and health outcomes for children and adolescents (American Academy of Pediatrics, n.d.-a). Having access to and utilization of pediatric medical homes is associated with greater discretionary healthcare utilization including preventive visits, improved general health of the child, better health-promoting behaviors, and reduced utilization of unnecessary healthcare services including emergency department visits and hospital stays (American Academy of Pediatrics, n.d.-b; Homer et al., 2008; Long et al., 2012). Although our findings appear to support medical homes and their roles in aiding mental health service utilization, medical homes have demonstrated mixed findings in the literature pertaining to their utility in both the utilization of mental health services and other health services, with only a few examining pediatric populations. For example, among adult populations, one study found that among patients who received care consistent with the definition of a patient-centered medical home (PCMH) and those who did not, there were no significant differences in healthcare utilization or expenditures (Bowdoin et al., 2018). However, Jones et al. (2015) report that patients with a PCMH were more likely than those without a usual provider to visit a mental health specialist or receive mental health counseling. Another study found that women and children were engaged in behavioral health care at greater rates within facilities classified as PCMHs compared to traditional settings (Abu-Ghname et al., 2019).

This study found some evidence that insurance coverage for mental healthcare needs is an important factor for treatment access. Comparison of frequencies and weighted percentages between the groups that perceived no problems accessing mental healthcare and those that perceived difficulty accessing care showed that those whose insurance did not cover mental health services or only sometimes covered such services were considerably more likely to report barriers to care access. Similarly, insurance coverage has demonstrated associations in other studies pertaining to access to mental health services (Kataoka et al., 2002; Triplett et al., 2022). The financial cost of mental health care has long been a barrier even for those with adequate insurance coverage. Further, current evidence indicates that underlying structural inequalities determine, or impact, health insurance coverage, its consistency, and adequacy among children and adolescents (Soylu et al., 2018). Usually, children from low-income families and those from underserved or minority groups are more likely to be underinsured or have inconsistent health insurance coverage (Kushel et al., 2006; Ma et al., 2008; Soylu et al., 2018). This study adds evidence to the growing consensus that healthcare insurance coverage is one of the primary factors in determining access to and utilization of healthcare services among pediatric-aged children, including access to mental health care, well-child visits, and other preventive care services (Selden & Hudson, 2006).

A notable difference in our findings was that age was not found to be significantly associated with perceived difficulty accessing mental health care services, which is inconsistent with previous studies. Mental health service use was found to increase as children begin their teenage years, before stabilizing around the ages of 15 to 17. For example, in a study conducted by the Substance Abuse and Mental Health Services Administration (SAMHSA), in a sample of children between 12 and 13 years of age, health services usage was around 13.0%. In comparison, usage reached 14.1% in cohorts of children who were 14 to 15 years as well as in children aged 16 to 17 years (Lipari et al., 2016). These findings were corroborated by Ringeisen and et al. (2016), who found outpatient therapist and clinic service utilization increased for ages 12 to 14 from 9.0 to 11.0% before stabilizing at that rate in children aged 15 to 17 years (Ringeisen et al., 2016). We suggest that further research be conducted to discern differences in health care needs, usage, and access between age groups.

We found that children from households where the highest education level was less than a high school diploma had less perceived difficulty accessing mental health services compared to children from households with college graduate or more in education. Current evidence on the associations between parental educational attainment and their children’s health and healthcare utilization is mixed and inconsistent (Bøe et al., 2021; Wu & Qi, 2022). Some prior evidence indicates that, among children with greater healthcare needs, lower parental education level is associated with lower use of healthcare and higher rates of unmet care (Bitsko et al., 2022; Kuhlthau et al., 2004). In terms of perceived access, Wu and Qi (2022) argue that because educated parents have more access to health information, potentially higher health literacy and a tendency to utilize healthcare services more efficiently, they are more likely to generate different child health outcomes and have different opinions towards perceived and actual access to healthcare services. Davis-Kean et al. (2021) stated that, despite correlations between different socioeconomic indicators, each tends to benefit child health in a unique way, as parental education level is a strong predictor of parental health beliefs and behaviors while their income shapes the provision of resources and materials (Davis-Kean et al., 2021). Other researchers have also indicated that more highly educated individuals are more likely to perceive suboptimal access to healthcare (Kasman & Badley, 2004).

On the other hand, Porterfield and McBride (2007) argue that parents will not seek access to a particular health service if they believe that their child does not need that service; hence, their perceptions shape actual service access. They further note that, among mothers of children with mental health conditions, those who do not complete high school have a substantially lower perceived need for specialized healthcare such as specialist physician services compared to mothers with a college degree (Porterfield & McBride, 2007). Therefore, parents with lower educational levels may not always recognize the importance or need for specific preventive healthcare or even be aware of the services provided––making them less likely to acknowledge potential barriers to accessing these services (Porterfield & McBride, 2007). Thus, in the current study, education can be considered as a unique factor in shaping perceptions of access to mental health care services.

Meeting criteria for having a medical home and insurance coverage were two enabling factors that contributed to less perceived difficulties in obtaining mental health services for their children. These are two well-known and prevalent hurdles for many families. While positive strides have been made in addressing them through efforts that integrate mental health care into primary care (Cole et al., 2019; Funk et al., 2008; Lund et al., 2016), this often only partially addresses the problem as those from low-income backgrounds still experience lower rates of engagement and greater rates of attrition in medical home coverage (Cole et al., 2019; Warden et al., 2009). Even those who manage to utilize mental health services may not attain the same amount of effectiveness in comparison to those from higher income backgrounds (Eamon & Venkataraman, 2003). Thus, implementing strategies that account for relevant socioeconomic determinants that these families experience may contribute to improved measures of patient engagement and therapeutic benefits.

Despite the relevance of our findings for public health and health policy, the results should be interpreted considering several limitations. Due to the nature of the NSCH data, our cross-sectional analysis makes it difficult to assess the causality in or directionality of any potential associations between predisposing, enabling, and need factors and perceived mental health service access. Further, the nature of a cross-sectional analysis is susceptible to recall and non-response bias from the survey population. Finally, certain variables had at random missing data due to inadequate responses from the participants, which contributed to a reduction in the statistical power and representativeness of our sample. These missing values could potentially complicate the interpretation of the results or could have introduced bias into the analysis. While we acknowledge the limitations of this research, the nature of this analysis may be helpful in piquing interest in pursuit of further related studies. We hope that our colleagues will engage with the questions raised in this study and carry on with meaningful investigations into the crisis of child and adolescent mental health and health care in the US.

## Conclusion

This study aimed to address current gaps in the literature regarding barriers and facilitators that contribute to disparities in accessing mental health services among children. Through analysis of nationally representative data of children from the 2020–2021 National Survey of Children’s Health, this study provides additional evidence about various factors that contribute to difficulties in accessing mental health services. It also adds to the growing knowledge concerning the role and benefits of medical homes as well as availability and quality of health insurance coverage in the realm of mental health care. Future research is needed to further elucidate relationships between the variables we examined, potentially through a mixed-methods design which offers the opportunity to simultaneously conduct qualitative and quantitative analyses into potential variables of interest. This could lead to additional qualitative data with queries about the specific mental health services that were sought by patients and their experiences with these services. In addition, addressing the social and economic determinants of healthcare access and other structural barriers such as financial abilities and insurance coverage through policy changes can serve to improve outcomes in regard to perceived or realized access to and utilization of mental health services in the future (Gnanapragasam et al., 2023). Despite needs for further evidence in the investigated subject, future policies and programs aiming to reduce or eliminate disparities in access to services among children with mental health disorders should design more comprehensive, child-centered, and culturally appropriate interventions with specific emphasis on children living in poverty, those with limited or no health insurance coverage, and children of racial and ethnic minoritized backgrounds.

Of particular importance, schools can be an ideal place for effective mental health program and policy implementation to potentially overcome a few structural barriers to mental health care among children and adolescents with or without mental health conditions. Schools have long been considered vital sources of mental health treatment for many children seeking these services (Suiter & Meadows, 2023). Most schools in the US provide some basic health services, and they have long been considered as a suitable place to mitigate some of the barriers in accessing health and healthcare, including mental health care services (Suiter & Meadows, 2023). In 2016, the National Academy of Pediatrics recommended that schools should have at least one full-time registered nurse tasked with monitoring the biopsychosocial wellness of students (Holmes et al., 2016). However, given insufficient funding levels for mental and behavioral health services in many US school systems, significant new investment would be required to make this recommendation a reality (Komisarow, 2022). As such, the combination of increased vulnerability and need for mental health services by older teenagers coinciding with a lack of availability also contributes to difficulties in obtaining care. Additionally, while the problem with adequate funding is common to both, disparities in this area often leave child and adolescent services with fewer resources than their counterparts (Borah et al., 2021; McGorry, 2007).

## Supplementary Information

Below is the link to the electronic supplementary material.

Supplementary file1 (DOCX 22 KB)
