# Supplementary material for: Disparities in Access to Mental Health Services Among Children Diagnosed with Anxiety and Depression in the United States
Source: Community Ment Health J. 2024 Jun 22;60(8):1532–46. doi: 10.1007/s10597-024-01305-3 (PMC11579094; doi:10.1007/s10597-024-01305-3)
Supplement: Supplementary file 1 — Supplementary file1 (DOCX 22 KB) [file 10597_2024_1305_MOESM1_ESM.docx]

**Supplementary Table 1**. Subgroup analysis modeling the associations between predisposing, enabling, and need factors of perceived access to child’s mental health services according to caregivers (children with diagnosed anxiety only, n=3,250, NSCH, 2019-2020, the United States)

| Characteristics | Adjusted OR (95% CI) |
| --- | --- |
| **Predisposing Factors** |  |
| Age groups (ref: 6-11 years) |  |
| 12-17 years | 0.712 (0.51, 1.01) |
| Sex (ref: female) |  |
| Male |  |
| Race and ethnicity (ref: non-Hispanic White) |  |
| Hispanic | 0.77 (0.44, 1.36) |
| Non-Hispanic Black | 0.68 (0.35, 1.32) |
| Non-Hispanic Asian and others | 0.77 (0.41, 1.41) |
| Family structure (ref: single parent) |  |
| Two parents (currently married or unmarried) | 0.77 (0.50, 1.18) |
| Grandparent household or other | 0.65 (0.32, 1.32) |
| **Enabling Factors** |  |
| Highest household education level (ref: college graduate or more) |  |
| Less than high school | 0.30 (0.10, 1.36) |
| High school ^a^ | 0.56 (0.31, 1.01) |
| Some college | 0.81 (0.51, 1.28) |
| Primary household language (ref: English) |  |
| Other than English | 0.87 (0.29, 2.63) |
| Household poverty status (ref:<99% FPL) |  |
| 100-199% FPL | 1.11 (0.58, 2.12) |
| 200-399% FPL | 0.73 (0.38, 1.41) |
| ≥400% FPL | 1.01 (0.53, 1.94) |
| Medical home (ref: no) ^b^ | 0.38 (0.28, 0.52)‡ |
| Consistency of insurance coverage (ref: currently uninsured or had gaps in coverage in the past 12 months) |  |
| Insured all past 12 months | 1.44 (0.60, 3.44) |
| Insurance coverage for mental or behavioral health needs (ref: sometimes or never) |  |
| Always | 0.18 (0.12, 0.26)‡ |
| Usually | 0.32 (0.21, 0.48)‡ |
| Uninsured | 0.76 (0.36, 1.60) |
| Usual place for health care/advice (ref: no) |  |
| Yes | 1.05 (0.56, 1.95) |
| **Caregiver Need Factors** |  |
| Caregiver’s self-rated physical health status (ref: not good) |  |
| Good | 0.88 (0.51, 1.52) |
| Caregiver's self-rated mental or emotional health (ref: not good) |  |
| Good | 0.92 (0.55, 1.54) |
| **Child Need Factors** |  |
| General health status of child (ref: fair or poor) |  |
| Excellent or very good | 0.88 (0.33, 2.34) |
| Good | 1.12 (0.39, 3.16) |
| *Abbreviations*: OR, Odds Ratio; CI, Confidence Interval; ref, reference; FPL, Federal Poverty Level | |
| * p-value <0.05; †p-value<0.01; ‡p-value<0.001. | |
| ^a^ Including vocational, trade, or business schools. | |
| ^b^ Whether the child meets the criteria for having a medical home. | |

**Supplementary Table 2.** Subgroup analysis modeling the associations between predisposing, enabling, and need factors of perceived access to child’s mental health services according to caregivers (children with diagnosed comorbid anxiety and depression, n=2,848, NSCH, 2019-2020, the United States)

| Characteristics | Adjusted OR (95% CI) |
| --- | --- |
| **Predisposing Factors** |  |
| Age groups (ref: 6-11 years) |  |
| 12-17 years | 1.00 (0.66, 1.53) |
| Sex (ref: female) |  |
| Male | 1.20 (0.87, 1.66) |
| Race and ethnicity (ref: non-Hispanic White) |  |
| Hispanic | 1.35 (0.86, 2.14) |
| Non-Hispanic Black | 4.18 (2.13, 8.23)‡ |
| Non-Hispanic Asian and others | 1.60 (1.02, 2.54)* |
| Family structure (ref: single parent) |  |
| Two parents (currently married or unmarried) | 1.29 (0.91, 1.83) |
| Grandparent household or other | 0.91 (0.52, 1.60) |
| **Enabling Factors** |  |
| Highest household education level (ref: college graduate or more) |  |
| Less than high school | 0.44 (0.14, 1.33) |
| High school ^a^ | 0.81 (0.50, 1.29) |
| Some college | 0.96 (0.64, 1.44) |
| Primary household language (ref: English) |  |
| Other than English | 2.70 (0.75, 9.67) |
| Household poverty status (ref:<99% FPL) |  |
| 100-199% FPL | 0.92 (0.56, 1.50) |
| 200-399% FPL | 1.00 (0.61, 1.65) |
| ≥400% FPL | 1.01 (0.59, 1.74) |
| Medical home (ref: no) ^b^ | 0.32 (0.23, 0.45)‡ |
| Consistency of insurance coverage (ref: currently uninsured or had gaps in coverage in the past 12 months) |  |
| Insured all past 12 months | 0.67 (0.33, 1.39) |
| Insurance coverage for mental or behavioral health needs (ref: sometimes or never) |  |
| Always | 0.17 (0.11, 0.25)‡ |
| Usually | 0.39 (0.26, 0.60)‡ |
| Uninsured | 0.49 (0.23, 1.05) |
| Usual place for health care/advice (ref: no) |  |
| Yes | 2.41 (0.52, 3.85) |
| **Caregiver Need Factors** |  |
| Caregiver’s self-rated physical health status (ref: not good) |  |
| Good | 1.02 (0.65, 1.61) |
| Caregiver's self-rated mental or emotional health (ref: not good) |  |
| Good | 0.93 (0.61, 1.40) |
| **Child Need Factors** |  |
| General health status of child (ref: fair or poor) |  |
| Excellent or very good | 0.57 (0.32, 1.01) |
| Good | 1.07 (0.57, 2.02) |
| *Abbreviations*: OR, Odds Ratio; CI, Confidence Interval; ref, reference; FPL, Federal Poverty Level | |
| * p-value <0.05; †p-value<0.01; ‡p-value<0.001. | |
| ^a^ Including vocational, trade, or business schools. | |
| ^b^ Whether the child meets the criteria for having a medical home. | |
